# Tailoring the Random Lasing Properties by Controlled Phase Separation Process in PMMA:PVK Dye-Doped Polymeric Blends

**DOI:** 10.3390/polym13183182

**Published:** 2021-09-19

**Authors:** Konrad Cyprych, Lech Sznitko

**Affiliations:** Advanced Materials Engineering and Modeling Group, Wroclaw University of Science and Technology, Wybrzeze Wyspianskiego 27, 50-370 Wroclaw, Poland; konrad.cyprych@pwr.edu.pl

**Keywords:** random laser, dye laser, polymeric blend, double-phase system, morphology, phase separation, waveguiding, quasi-waveguiding, PVK, PMMA, rhodamine 6g

## Abstract

This article describes the random lasing (RL) phenomenon obtained in a dye-doped, polymeric double-phase system composed of PMMA and PVK polymers. It shows how relative concentrations between mentioned macromolecules can influence lasing parameters of the resulting blends, including obtained emission spectra and threshold conditions. We describe the influence of lasers’ composition on their morphologies and link them with particular RL properties. Our studies reveal that the disorder caused by phase separation can support the RL phenomenon both in the waveguiding and quasi-waveguiding regimes. Changing the relative concentration of polymers enables one to switch between both regimes, which significantly influences threshold conditions, spectral shift, number of lasing modes, and ability to support extended and/or localized modes. Finally, we show that a simple phase separation technique can be used to fabricate efficient materials for RL. Moreover, it enables the tailoring of lasing properties of materials in a relatively wide range at the stage of the laser material fabrication process in a simple way. Therefore, this technique can be seen as a fast, cheap, and easy to perform way of random lasers fabrication.

## 1. Introduction

Mixing different polymers into one bulky material is a strategy that allows the design of novel types of media that will gain new physical properties or functionalities. Contrary to the chemical synthesis, which aims to develop entirely new compounds, the mentioned approach utilizes already known components. The drawback of this attempt is that most polymers are not miscible in each other, and hence, they frequently form multi-phase systems. However, for some applications, the consequences coming from phase separation might be very beneficial. For example, it can be responsible for diffusive-like scattering; thus, polymeric blends can find applications as diffusers used for lighting purposes and display technologies [1,2]. Moreover, the interface between separated phases is mainly characterized by rather a high surface area. This feature is applied to improve the operation performances of bulk heterojunction polymeric solar cells [3,4,5,6], organic photodetectors [3,7], organic transistors [8,9], and organic light-emitting diodes [3,10].

As was discussed before, phase separation can lead to a significant boost of light scattering. This effect stands behind the so-called random lasing (RL) phenomenon. Random lasers are optical devices that utilize multiple light scattering to obtain positive feedback [11], contrary to classical lasers where an external optical resonator is used for this purpose. Many types and designs of random lasers were developed. For example, Lawandy et al. have proposed to use so-called laser paints, where scatters are added to the volume of a liquid solution of organic laser dye [12]. The scatterers could be made of neutral inorganic TiO_2_ nanoparticles (or similar) [12,13] or metallic particles supporting the surface plasmon resonance to enhance the local electric field [14,15,16]. Similar strategies could be applied to modify solid-state polymeric matrices containing laser dyes or semiconducting polymers [17,18,19,20,21]. Alternatively, the colloidal quantum dots can be applied as emitters [22,23]. More complex systems can utilize photonic glasses [24] or polymer/liquid crystalline two-phase systems [25,26]. The effect of RL could also be obtained in single-phase systems, where high surface roughness is responsible for light scattering [27,28,29] or in fine grind laser crystals, where reflection from crystals facets are establishing the multiple light scattering, e.g., as in the case of ZnO powders [30] or organic dye crystals [31].

As the principle of RL is based on disorder, organic matter seems to be perfect for this role. The undoubted advantage of organic matter is the easiness of processing, using many different techniques. Such materials can be coated using spin-coating, doctor blade, casting techniques [32], etc., or fabricated as nano or microfibers in the process of electrospinning [33,34]. In general, almost all of the so-called wet processing techniques can be applied to fabricate random lasers. For example, Liao and coworkers have shown that polymeric random lasers can be easily printed using inkjet printers [35] or 3D printers [36]. More bizarre techniques, more suitable for biomaterials processing, can even involve such techniques as cooking. For example, this approach was used to produce random lasers in the form of protein skins made from amyloid nanofibrils [37]. Finally, different organic phases such as polymers and liquid crystals can be mixed together, as in the case of electrically controlled random laser generating white laser emission [26].

In this article, we investigate the composition influence of polymeric blends made of two non-miscible polymers, poly(methyl methacrylate) (PMMA), and poly(9-vinylcarbazole) (PVK), functionalized with rhodamine 6g laser dye on RL performance. Both polymers were selected for our studies because they are not mixing and are characterized by entirely different refractive index values ($n_{pmma}=1.49;n_{pvk}=1.69$). As we are targeting to obtain RL, which requires multiple light scattering, the high refractive index contrast will assure a high scattering cross-section. Moreover, both polymers and rhodamine 6g laser dye can be dissolved in chloroform. Hence, selected materials are perfect for preparing liquid solutions of polymers with relative concentrations spanning a wide range of values and suitable to such wet-processing techniques as spin-coating.

It is worth noting that purely polymeric two-phase systems were not extensively investigated according to the generation of light in the process of RL. Some attempts to this issue were carried out by Zhao and coworkers who have obtained RL in dye-doped polystyrene (PS) and PMMA blends [38]. Cerdan and coworkers [28] managed to obtain RL supported by surface roughness induced by phase separation; however, they investigated hybrid organic/inorganic blends composed of poly(2-hydroxyethyl methacrylate) (pHEMA) and octa(hydroxypropyldimethylsilyl)-POSS (8OH-POSS). Alternatively, Wetter et al. [39] have shown RL in rhodamine 6g silica aerogel. All mentioned studies were carried out in similar pumping conditions as described here, but only the first-mentioned were referring to purely polymeric systems. Therefore, the topic of phase separation between two or more polymers and its influence on RL has not yet been explored in detail.

Moreover, so-called block-copolymers are capable of creating highly ordered structures during the phase-separation process. It was already evidenced in the literature that such structures can be used for photonics [40]. In such a case, the spatial ordering is precise enough to fabricate distributed Bragg reflector (DBR) lasers [40,41]. If we consider the features mentioned above of multi-phase polymeric systems, it is more surprising that the topic of such random lasers is somewhat omitted in the literature.

## 2. Materials and Methods

### 2.1. Preparation of Samples

At first, liquid solutions of PMMA (Sigma Aldrich, Saint Louis, MO, USA) and PVK (Sigma Aldrich, Saint Louis, MO, USA) polymers were prepared. The PMMA was characterized by molecular weight Mw = 150 kDa and PVK by Mw = 72 kDa. Both were dissolved in chloroform (Sigma Aldrich, Saint Louis, MO, USA) in separate vials. After that, a proper volume of rhodamine 6g (Photonic Solutions Ltd, Edinburgh, United Kingdom) and chloroform mixture was added to each vial to form solutions of polymers concentration equal to 30 mg/mL and dye to polymer dry mass ratio equal to 2%. When all components were completely dissolved, stock solutions were mixed in the volumetric (PMMA to PVK) ratios, as shown in Table 1. We selected a wide range of mixing ratios to visualize the changes that most strongly affect the sample morphology. For the sake of simplicity, we will use the percentage of volumetric content of PVK used for samples preparation in their liquid forms to refer to a particular sample. PVK, as well as PMMA contents, are also presented in Table 1.

Samples in the form of asymmetric slab waveguides were fabricated using a spin-coating technique. For this purpose, we used microscopic glass slides (Thermo Fisher Scientific, Waltham, MA, USA) as substrates for layers deposition. Each substrate was previously cut to have a rectangular shape of 26×26 mm^2^, cleaned in the ultrasonic bath for 10 min in detergent solution at 50 degrees Celsius, and then rinsed with distilled water. This procedure ensured additional cleaning and removal of most of the dirtiness left on substrates after the cutting procedure. After drying, substrates were placed into a POLOS 150i (SPS-Europe B.V., Putten, Netherlands) spin-coater to start the layers fabrication process. Before the spinning was initiated, the 200 µL of polymer and dye mixture was deposited on the substrate using SBS-PIP100 automatic pipette (Steinberg Systems, Hamburg, Germany). Next, the coating process was initialized. The first stage took 20 sec, the rotation speed was equal to 500 rpm, while the second stage was carried out at 1000 rpm for 120 sec. When spinning was completed, the samples were stored for more than 48h in the dark before further spectroscopic and morphological studies were carried out. The scheme of sample preparation is shown in Figure S1.

### 2.2. Morphological Studies

Light microscopy was used to investigate the morphologies of obtained layers. For this purpose, we used IX71 (Olympus, Shinjuku, Japan) microscope operating in reflection mode and the Dimension V (Veeco, Plainview, New York, NY, USA) atomic force microscope (AFM) operating in tapping mode. The first technique was used to determine morphological changes at the micrometer-scale level. It provided a fast and rough estimation of samples morphologies. The insight into the morphology at the nanoscale level was assured by AFM microscopy. This technique allowed us to obtain images of high resolution, up to several nanometers; however, this technique is relatively slow and prone to distortions if scanning is fast and height amplitudes are significant. Both techniques are complementary to the research described in this article.

The thicknesses of layers were determined at the end of all measurements using a Dektak 3 (Veeco, Plainview, New York, NY, USA) profilometer. In this case, central regions of layers were removed using a scalpel to expose the surfaces of glass slides, and then the profilometer measured the depth of such a formed scratch.

### 2.3. Random Lasing

RL measurements were carried out in an experimental setup comprising the following optical devices and elements: a nanosecond Nd:YAG laser Surelite II (Continuum Lasers, San Jose, California, CA, USA) generating a laser light of wavelength equal to 355 nm (third harmonic) with a repetition rate of 10 Hz, pulse duration 5 ns, and energy in pulse 156 mJ, the Horizon I (Continuum Lasers, San Jose, California, CA, USA) optical parametric oscillator (OPO) capable of tuning the laser light wavelength between 192 and 2750 nm in the parametric three-wave mixing process, and the optical system consisting of an achromatic half-wave plate, achromatic Glan laser polarizer, afocal system made of two convex lenses with magnification 5×, adjustable slit, and a cylindrical lens. The laser light from OPO was used to excite polymeric samples; it was adjusted to the wavelength of 535 nm to match the absorption band of rhodamine 6g laser dye [42]. According to the Malus law principle, a half-wave plate and the Glan laser polarizer were used to control the incident beam energy. Adjustable slit and cylindrical lens were used to form a horizontally oriented stripe shape area of excitation of 3.91 mm width and 0.64 mm height. The emission was acquired from samples edges, perpendicular to the polarization and direction of excitation light propagation, using the Shamrock SR 163 (Andor Technology Ltd, Belfast, Northern Ireland) high-resolution Czerny–Turner fiber spectrometer equipped with the iVac (Andor Technology Ltd, Belfast, Northern Ireland) CCD camera. The scheme of the experimental setup is presented in Figure S2.

## 3. Results and Discussion

### 3.1. Morphology

**Figure 1:**
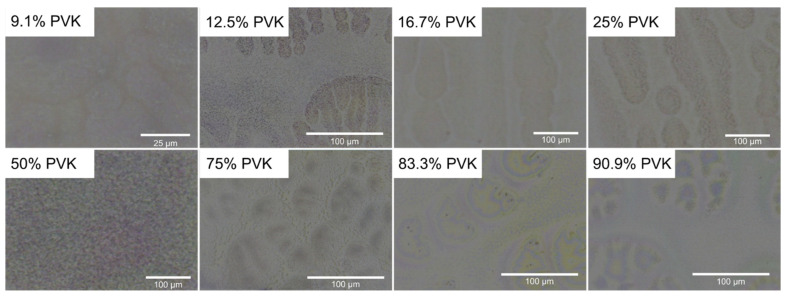
Light microscope images (reflection mode) of samples containing 9.1%, 12.5%, 16.7%, 25%, 50%, 75%, 83.3%, and 90.9% of PVK content.

At first, morphologies of fabricated samples were investigated using light microscopy. Exemplary images of layers morphologies obtained for different concentrations of PVK in resulting polymeric blends are shown in Figure 1. The granular structure at the micrometer scale is visible for the concentrations of PVK ranging between 9.1 to 50%. For higher concentrations, especially easily visible for PVK content equal to 75%, the micrometer size structures are characterized by holes instead of the granules that were observed for lower loads of PVK. It indicates that, for PVK concentrations below 75%, the dominating phase is a PMMA rich phase (we will call it later PMMA framework) in which the PVK particles are suspended. For the PVK concentrations equal to 75% and higher, the dominating phase is PVK rich phase (we will later call it PVK framework), while small PMMA rich areas are visible as holes in polymeric blends. The 50% PVK mixture presents a blend with an indistinguishable composition. The others have the characteristic features of several tens to hundreds of micrometers. Some of them are characterized by elongated shapes and are composed of polymeric particles of bigger sizes (PVK concentration between 9 and 25%). They possibly appeared due to the immobilization of PVK particles at nucleation sites, which did not allow bigger ones to be centrifuged from the substrates during the spin coating process. Similar structures can be observed for samples with PVK concentrations equal to 75% and higher; however, they are smaller and more complex in shape. The reason for their occurrence is similar to the previous case, but here, the nucleation sites might be responsible for the formation of a thicker PVK framework. Thus, the features are somewhat different from those observed for lower PVK loads.

**Figure 2:**
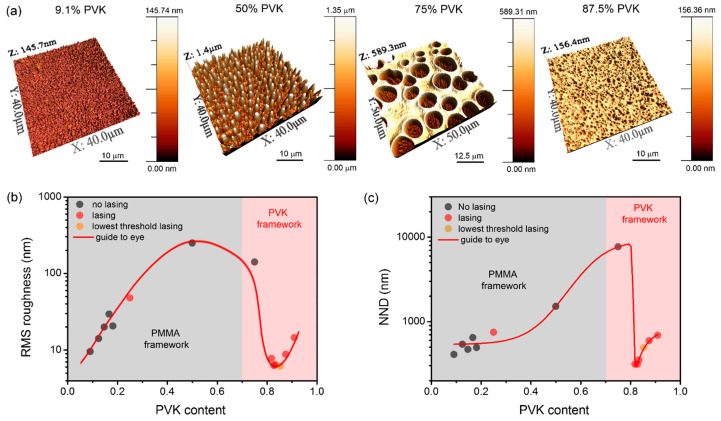
(**a**) A comparison of PMMA:PVK blend morphologies obtained in AFM measurements for different relative polymers concentrations, namely: 9.1, 50, 75, and 87.5% of PVK; (**b**) the dependency of roughness (calculated as RMS deviation from average height) on PVK concentration in PMMA:PVK blends; (**c**) the dependency of average nearest neighbor distance (NND) between hills or holes on PVK concentration.

A more detailed insight into blend morphologies is possible thanks to the AFM measurements. Figure 2a shows four exemplary images obtained using the AFM technique for samples loaded with PVK in the concentration of 9.1, 50, 75, 90.9%. The morphologies of all investigated samples are shown in supplementary info in Figure S3a.

For low PVK concentrations, the surface structure is dominated by granular features (with round symmetry) of a few tens of nanometers in height and below one micrometer in diameter (see Figure S3b). The increase in PVK content results in the growth of the granules’ height and width, even up to a micrometer scale for the concentration of PVK equal to 50%. The enlargement of surface roughness (understood as root mean square (RMS) deviation from the average height of the probed surface) accompanies the increase in PVK content until the concentration crosses 50% of the PVK amount. The RMS roughness was calculated using a roughness analysis protocol available in WSxM 5.0 software [43]. The function of RMS roughness vs. PVK content is shown in Figure 2b. In the mentioned PVK concentration range, the roughness changes from 9.6 nm (9.1% of PVK) to 248.6 nm (50% of PVK). When the concentration of PVK reaches 75%, the morphology is entirely changed. The PVK granules merge into one polymeric framework, containing PMMA domains in the form of round-shaped depressions. Interestingly, the PMMA phase can still contain some smaller domains, possibly made of PVK particles, which are visualized as spikes inside the holes in Figure 2a and Figure S3c. Even if the whole structure is highly complex, RMS roughness is decreasing in such cases to 141.0 nm, mainly due to the high contribution of the relatively flat PVK framework. The further increase in PVK results in the formation of nearly flat surfaces with some hole-like features of tens of nanometers depth and below a micrometer in diameter (see Figure 2a and Figure S3a,d). The decrease in RMS roughness in such a case is even more rapid. For the concentration of PVK equal to 81.8%, it takes the value of 7.8 nm. When PVK concentration is equal to 85.3%, the roughness is minimal (RMS roughness = 6.2 nm). For higher concentrations, roughness rises again, reaching the value of 14.5 nm for 90.9% of PVK. The changes in roughness are relatively low for the range of PVK concentrations spanning between 81.8% and 90.9%. A slight increase in RMS can be observed for higher PVK concentrations. It could be explained in terms of deeper holes resulting from lower PMMA concentration, which boosts the resulting roughness. On the other hand, a lower amount of PMMA is responsible for longer distances between holes and their smaller diameters.

Therefore, the resulting roughness value is a consequence of the interplay between the depth of holes and part of the area that they occupy in the scanned region of the sample. Hence, the roughness has to have minimal value for a particular PVK concentration, which is not the highest one as intuitively could be assumed.

**Figure 3:**
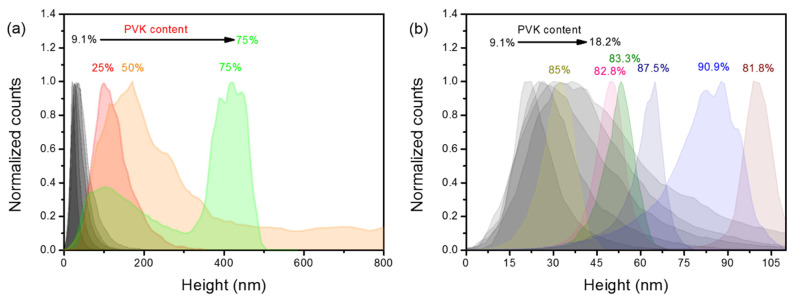
Normalized height histograms for different concentrations of PVK in PMMA:PVK blends; (**a**) the histograms for an increasing amount of PVK from 9.1 to 75%; (**b**) the comparison of height histograms for samples containing 9.1–18.2 and 81.8–90.9% of PVK.

In Figure 3a,b, we depicted the normalized height histograms taken from the areas shown in Figure S3a. For relatively low PVK concentrations (between 9.1 and 18.2%), the histograms are quite similar (semi-black distributions on Figure 3a,b). All of them show positive skewness, indicating the character of log-normal distributions. The right side shoulder becomes wider with the increase in PVK concentration, and the most probable height shifts towards greater values. In general, for the mentioned range of PVK concentrations, all distributions increase their width with growing PVK content, which is reflected in the higher RMS roughness parameter. The histogram is still quite similar for the 25% PVK concentration, but it is heavily shifted towards higher height values. For 50% of PVK, the right tail of the distribution does not reach the 0 value. It means that another distribution positioned at greater height values emerges (Figure 3a, the orange histogram). For the 75% PVK content, the height distribution becomes purely bimodal. It comprises two distributions: with lower maximal value and positive skewness; and quasi-symmetric distribution with higher maximal value (Figure 3a, the green histogram). Possibly, both distributions are responsible for different phases of polymers. For pits made of PMMA with PVK granules (see Figure S3a,c), the histogram is taking lower height values and shapes of positive skewness, while for the PVK framework, height values are much greater, and the distribution is relatively symmetric (with no skewness). The further increase in PVK content is shifting height histograms towards lower values as the holes-like structures become more shallow than in the previous case. Moreover, the shape of histograms is affected by negative skewness. The comparison between samples containing 12.5 and 83.3% of PVK is shown in Figure S4. It is clearly visible that both distributions have the opposite skewness, and both almost look like their mirror images. When the concentration of PVK exceeds 75%, the position of the most probable height value function vs. PVK content is not clear cut. For PVK contents varying from 81.8 to 85.3%, the height distribution is shifted to lower values. However, for concentrations equal to 87.5 and 90.9%, the most probable height value is once again rising.

As our research targets the optical properties of polymeric blends, another critical parameter for light waveguiding and scattering effect is the average nearest neighbor distance (NND) between holes or hills. This parameter can determine the shortest average distance that light has to travel inside the layer to experience the scattering event. For that purpose, we have utilized the flooding protocol available in WSxM 5.0 software [43] and applied it for AFM images shown in Figure S3a. The flooding level for each sample was selected in such a way that the NND parameter was showing the lowest possible value. The flooding maps with perimeters of hills and holes are presented in Figure S5. The evolution of NND with increasing PVK content is shown in Figure 2c. As can be seen, when granules of PVK become bigger (rising PVK content), the NND becomes greater. The NND increases from 409 nm for 9.1% of PVK up to 1511 nm for 50% of PVK. When the structure becomes dominated by the PVK framework and holes for 75% content of PVK, the NND is the greatest and reaches the value of 7675 nm. A further increase in PVK results in a sudden drop of NND to 312 nm for 81.8% of PVK. There are plenty of sites where small volumes of PMMA phase can emerge, visualized as holes in the PVK framework on AFM scans. Further increase in PVK and decrease in PMMA amount results in the increasing distance between the holes. The NND finally reaches a value equal to 688 nm for 90.9% of PVK.

### 3.2. Results of Random Lasing Measurements

RL measurements were carried out in the experimental setup described in the Methodology section.

**Figure 4:**
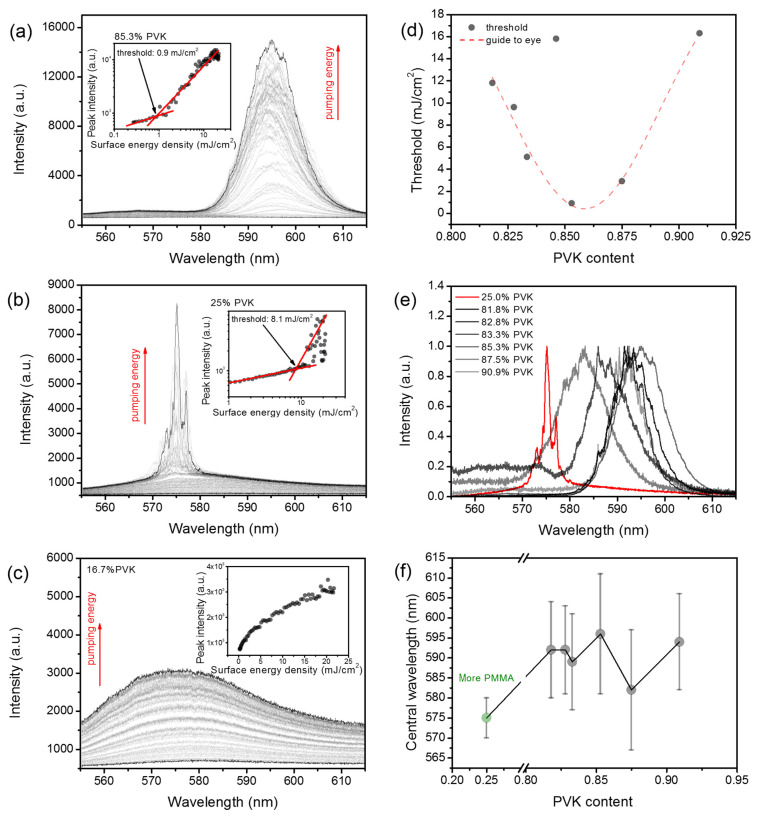
RL spectra, obtained for samples with 85.3% (**a**) and 25% (**b**) of PVK content and their evolution upon increasing pumping fluence. Insets are showing emission intensity dependencies on pumping fluences for both samples. Lasing thresholds are easily recognizable; (**c**) an example of spontaneous emission spectra and the evolution of its intensity in the function of pumping fluence **(inset)**, obtained for a sample with 16.7% of PVK; (**d**) the threshold dependency on PVK content; (**e**) a comparison of normalized RL spectra captured for different samples at the pumping fluence equal to 22 mJ/cm^2^; (**f**) the plot of the central wavelength of RL spectra as the function of PVK content. Error bars indicate RL bandwidths measured at the bottom of RL spectra.

Each fabricated sample was examined according to RL occurrence, but only some of them exhibited the mentioned phenomenon. The RL phenomenon has occurred mostly for blends containing a more significant amount of PVK. An exemplary evolution of the RL spectrum with increasing pumping fluence for the sample with 85.3% of PVK is shown in Figure 4a. The RL spectra are typically composed of many lasing modes randomly emerging from the gain profile [11]. The inset in Figure 4a shows a typical inflection point on emission intensity vs. pumping fluence plot, which is characteristic of the lasing threshold. The RL was observed for samples containing the following amount of PVK: 25, 81.8, 82.8, 83.3, 85.3, 87.5, and 90.9%. In most cases, for blends containing the more significant amount of PMMA, we have failed to observe the RL. Only one sample with 25% of PVK exhibited this phenomenon. The results for this sample are shown in Figure 4b. Typically, samples that were not evidenced to exhibit the RL showed spectra similar to those presented in Figure 4c. The intensity vs. pumping fluence plots for those samples showed no inflection points, and frequently, their slopes decreased with increased pumping, indicating that emission saturation occurred (inset in Figure 4c).

Importantly, the RL spectra obtained for samples with higher loads of PVK have shown substantial differences from the sample with higher PMMA load. Most of them are significantly red-shifted and broadened, and lasing modes are worse resolved. For this purpose, the threshold on PVK content dependency, shown in Figure 4d, is not containing the result obtained for a sample with 25% of PVK. As it can be seen, the threshold is dependent on PVK concentration, and it reaches the minimum equal to 0.9 mJ/cm^2^ for a PVK amount equal to 85.3%. The change in PVK content from the mentioned value results in the elevation of threshold conditions: for the sample with 90.9% of PVK, even up to 16.3 mJ/cm^2^; and for the sample with 81.8% of PVK, to 11.8 mJ/cm^2^.

As mentioned before, RL spectra for samples with a more significant amount of PVK differ a lot from the samples with the higher load of PMMA (see Figure 4e). The significant red-shift and increased overlapping between modes might be caused by emission waveguided in PVK dominated layers. As it is known, the PVK refractive index is around 1.69, while for PMMA, it is around 1.49. Therefore, if we assume the slab waveguide geometry of samples and consider that layers are deposited on glass slides with a refractive index of around 1.52, it is clear that samples with a higher amount of PMMA cannot support the waveguiding effect. So, the red-shift for higher loads of PVK might be due to the (i) re-absorption, (ii) occurrence of rhodamine 6g aggregates generated due to the possible lower solubility, or (iii) by the difference in polarity index of the PVK matrix with respect to PMMA. Strong red-shifting supports both latter mechanisms; however, the plot of the central wavelength position of RL band vs. PVK content shown in Figure 4f does not clearly confirm this hypothesis. We could expect that, for gradually changing polarity or decreasing solubility of rhodamine 6g, the observed red-shifting accompanied by the increase in PVK content is also gradual, but nothing like that happens. The red-shift of central wavelength seems to be rather step-like, from 575 nm, for PMMA rich samples, to 595 nm for samples heavily loaded with PVK. Both central wavelength and the bandwidth seem to fluctuate randomly, with the PVK amount above 81.8%. Therefore, the resulting RL spectra and lasing threshold conditions have to be mainly linked with the scattering-affected light-waveguiding effect. However, both mentioned earlier mechanisms of red-shifting could not be completely ruled out.

**Figure 5:**
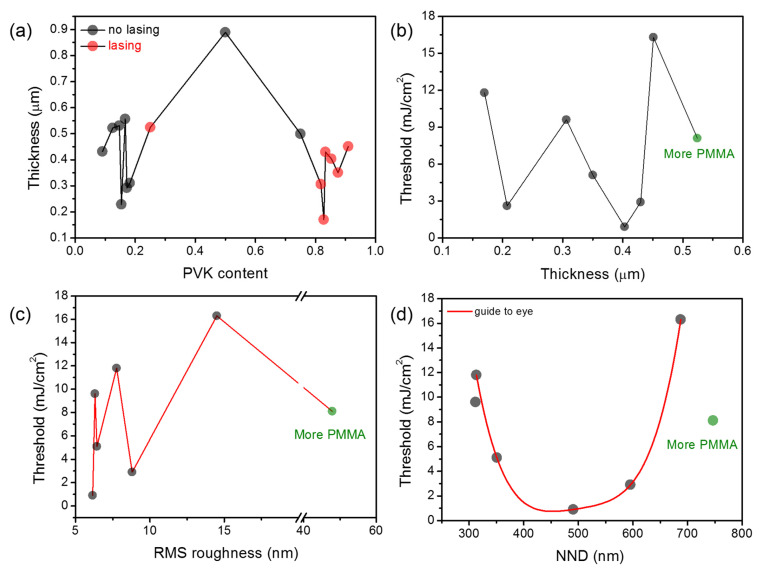
(**a**) The thicknesses of fabricated samples as the function of PVK content; (**b**) the threshold dependency on sample thickness; (**c**) the threshold dependency on RMS roughness; (**d**) the threshold dependency on NND between holes/hills.

An essential parameter affecting the waveguided modes in a slab waveguide is the layer thickness. Measurements of thicknesses were carried as described in the Methodology section using a profilometer. The obtained results, as functions of PVK content, are plotted in Figure 5a. As can be seen, the highest thickness is obtained for blends with 50% of PVK, and it decreases when a fraction of a particular polymer dominates. The results show that obtained layer thicknesses varied between 170 nm for the sample with 82.8% of PVK up to 889 nm for the sample with 50% of PVK; however, most of them span between around 0.2 to 0.5 μm. Such thicknesses are sufficient for the light waveguiding in the PVK matrix but insufficient for PMMA. Figure 5b shows the RL threshold as the function of layer thickness. Analyzing Figure 5b, we can conclude no correlation between the threshold and the sample thickness. The plot presented in Figure 2b shows that the threshold is the lowest for the sample with the lowest roughness. However, when this parameter is plotted vs. RMS roughness, it seems that there is no simple correlation between those two parameters (Figure 5c). Finally, a clear correlation can be found for threshold plotted vs. NND (Figure 5d). In this case, it is evident that the threshold decreases when NND approaches the value of c.a. 500 nm. As can be seen, the sample with a more significant amount of PMMA is out of this trend, as the mechanism of light waveguiding is possibly not involved in the light amplification process.

For samples with higher loads of PVK, the light can be waveguided and amplified in the polymeric framework made of PVK. Holes visible in the structure of polymeric blends are serving here just as scattering centers. The RMS roughness is due to the differences in height between PVK and PMMA phases; if the light is waveguided mostly in PVK polymer, the roughness influencing the propagation is somewhat different from that obtained in AFM measurements, which includes the contribution from both polymeric phases. Thus, the correlation between threshold and RMS is rather complex. Moreover, it is worth noting that RMS roughness for samples with PVK content varying between 81.8 and 90.9% is still very low. The scattering caused only by surface roughness will be less prominent than the scattering from phase boundaries. Therefore, for quasi-2D systems as considered here (thin layers), the lateral distance between scatterers will play the most important role in establishing the RL operation’s most relevant scattering conditions.

For a sample with lower PVK loads, the situation is entirely different. In general, the light cannot be waveguided in polymeric blend due to the low thickness and low refractive index compared to the substrate. It has to be considered that light will likely leak out to microscopic glass substrate. The emitted light collected by spectrometer from the edge of the sample will be mostly quasi-waveguided through the substrate. Therefore, it cannot reach strong enough enhancement, as the traveled distance by light in the active layer is too short to overcome the losses. However, when the roughness is high enough, the conditions for light waveguiding can locally change. Due to the high surface roughness, the light can be locally scattered to the active layer, and thus the light enhancement can be achieved in some specific regions of the sample. In other words, the high roughness can be locally responsible for coupling the light into the active layer, allowing the light to travel longer paths than would be possible for flat surfaces, or it can even help in establishing the light localization. Moreover, higher roughness means that larger spatial dimensions will characterize the PVK granules. Hence, such granules of relatively high refractive index can also serve as separate resonators. As the blend as a whole cannot support the waveguiding, the RL light from particular sites, where light amplification occurs, arrives at the spectrometer as outcoupled light guided through the substrate. This mechanism explains the blue-shifting of RL spectra for a sample with 25% of PVK. Moreover, relatively small sites capable of light enhancement can produce a relatively low number of localized modes; thus, the RL spectra are better resolved than those collected for higher PVK loads.

## 4. Conclusions

To conclude, we have conducted a detailed study on the influence of the morphology of double-phase polymeric systems on RL performance. To the best of our knowledge, the results of the research presented here are the first ones that concerned a wide range of relative concentrations of double-phase system components (PVK and PMMA) influence on the optically pumped RL occurrence. We have demonstrated that the RL phenomenon can be easily achievable when waveguiding conditions are met. Thus the RL phenomenon can be observed mainly for higher PVK concentrations, as the PVK polymer is characterized by a higher refractive index (1.69) than it is for the utilized substrates (1.52). For waveguiding conditions, the relative concentration between PMMA and PVK has a significant influence on lasing parameters. Particularly, it is possible to tune the threshold at the samples fabrication stage even by a factor of 18.1 in the range of concentrations spanning between 81.8 and 90.9% of PVK content. Moreover, extremely high scattering can also support the RL operation in a quasi-waveguiding regime for samples with a higher amount of PMMA (25% of PVK). However, in this case, more detailed studies concerning more precise changes in relative concentrations are required.

The obtained results show that the most crucial parameter for waveguided RL is the distance between scattering centers, which was approximately 500 nm in our case. For such quasi-2D waveguiding, the roughness plays a less important role, possibly leading to an increase in losses and thus the worsening of lasing performance. On the other hand, when quasi-waveguiding is the dominant mechanism of light propagation, high enough surface roughness enables light confinement and thus the coherent RL. Therefore, in the second regime, the optimized and relatively high roughness plays a positive role for RL.

The presented technique relying on phase separation enables the tailoring of lasing properties of materials in a relatively wide range of parameters. It can be used, in a simple way, at the stage of the laser material fabrication process and is suitable for most known wet processing techniques. Therefore, this technique can be seen as a fast, cheap, easy, and versatile method of random laser fabrication.

## Figures and Tables

**Table 1:** A list of investigated samples with information about PMMA to PVK volumetric ratios, PVK, and PMMA content.

**PMMA:PVK**	10:1	7:1	5.8:1	5:1	4.5:1	3:1	1:1	1:3	1:4.5	1:4.8	1:5	1:5.8	1:7	1:10
**PVK** **Content**	9.1%	12.5%	14.7%	16.7%	18.2%	25%	50%	75%	81.8%	82.8%	83.3%	85.3%	87.5%	90.9%
**PMMA Content**	90.9%	87.5%	85.3%	83.3%	81.8%	75%	50%	25%	18.2%	17.2%	16.7%	14.7%	12.5%	9.1%

## Data Availability

The data presented in this study are available on request from the corresponding author.

## Supplementary Materials

The following are available online at https://www.mdpi.com/article/10.3390/polym13183182/s1, Figure S1: Samples preparation scheme, Figure S2: Scheme of the experimental setup used for RL measurements, Figure S3: AFM images and surface profiles, Figure S4: Height histograms, Figure S5: Flooding maps.
