# Supplementary material for: Tailoring the Random Lasing Properties by Controlled Phase Separation Process in PMMA:PVK Dye-Doped Polymeric Blends
Source: Polymers (Basel). 2021 Sep 19;13(18):3182. doi: 10.3390/polym13183182 (PMC8467720; doi:10.3390/polym13183182)
Supplement: Supplementary file 1 [file polymers-13-03182-s001.zip › polymers-1374395-supplementary.pdf]

Supplement information

Tailoring the random lasing properties by controlled phase separation process in PMMA:PVK dye-doped polymeric blends

Konrad Cyprych and Lech Sznitko*

Advanced Materials Engineering and Modeling Group, Wroclaw University of Science and Technology, Wybrzeze Wyspianskiego 27, 50-370 Wroclaw, Poland

*Corresponding Author: lech.sznitko@pwr.edu.pl

Keywords: random laser, dye laser, polymeric blend, double-phase system, morphology, phase separation, waveguiding, quasi-waveguiding, PVK, PMMA, rhodamine 6G

Schemes of samples preparation and experimental setup used for random lasing experiments:

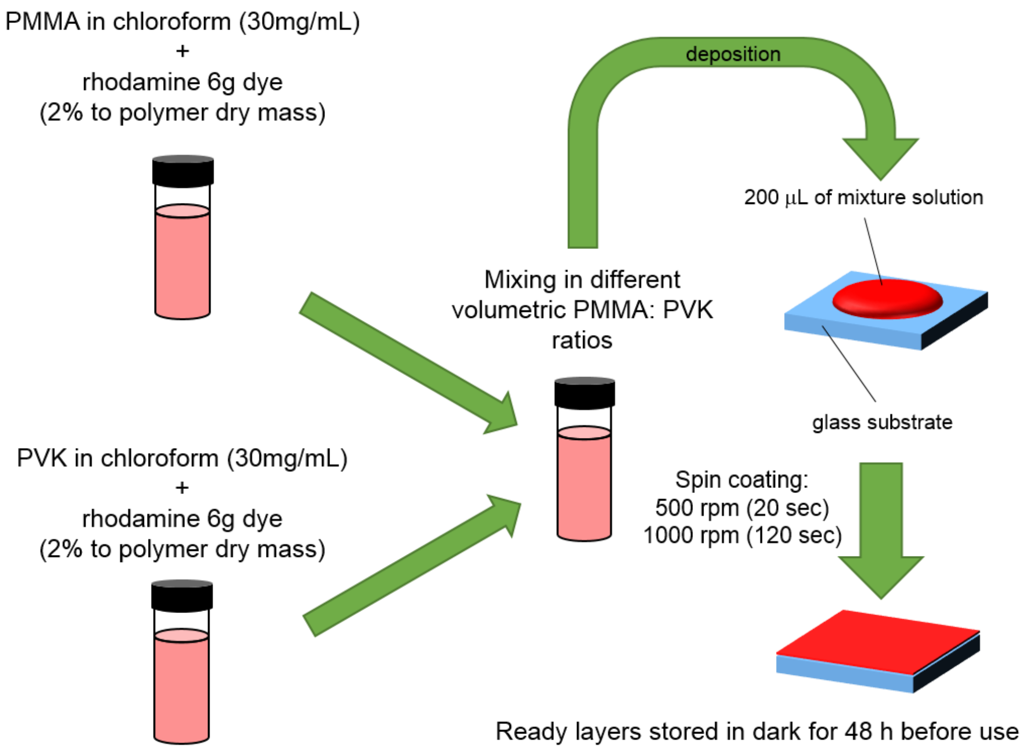

Figure S1. A scheme of the samples preparation procedure.

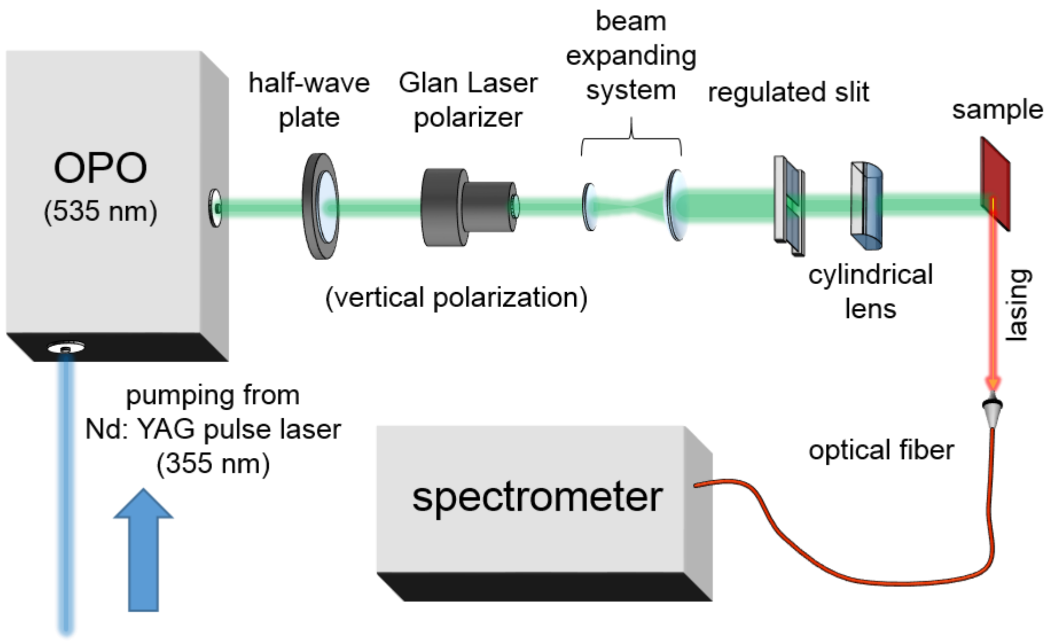

Figure S2. A scheme of the experimental setup used for random lasing experiments.

AFM images and surface profiles:

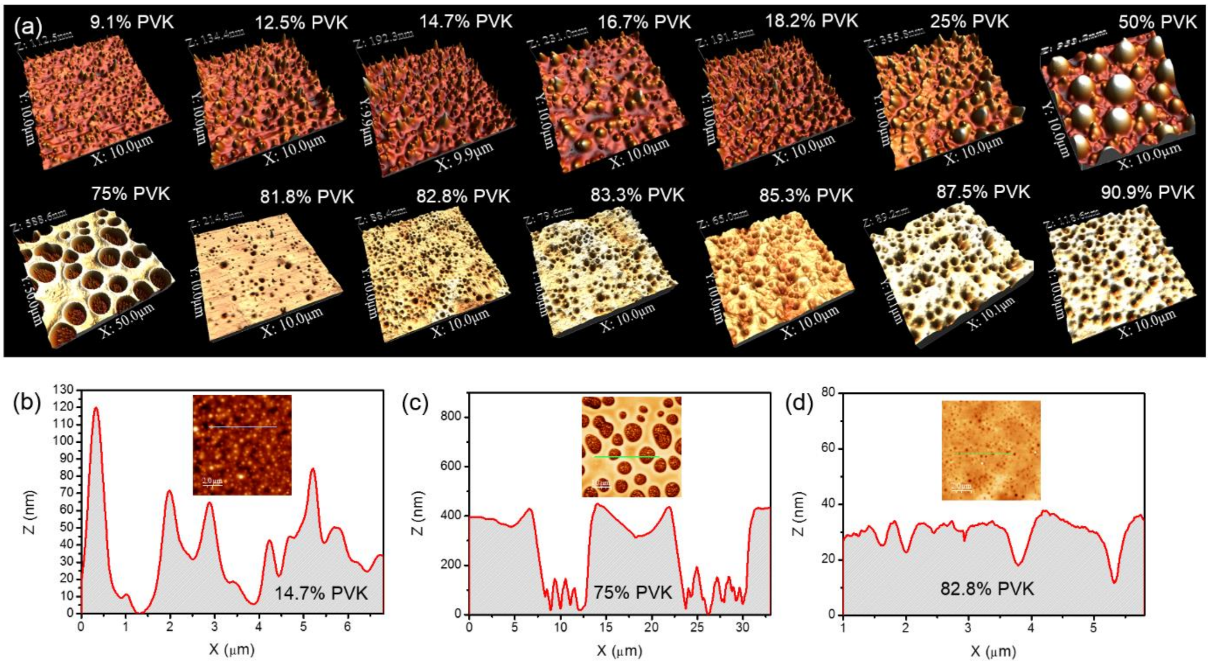

Figure S3. (a) 3D AFM images of PMMA:PVK blends with increasing PVK content. A comparison of exemplary surface profiles recorded for blends containing: **(b)** 14.7%; **(c)** 75%, and; **(d)** 82.8% of PVK.

Height histograms:

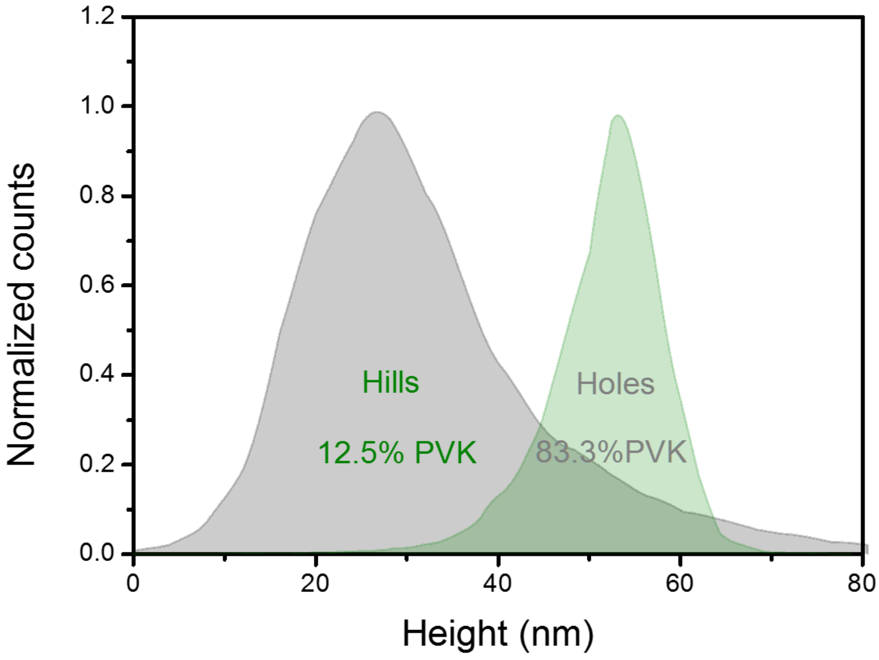

Figure S4. A comparison of height histograms shapes for samples dominated with hills (12.5% PVK) and holes (83.3% PVK).

Flooding maps

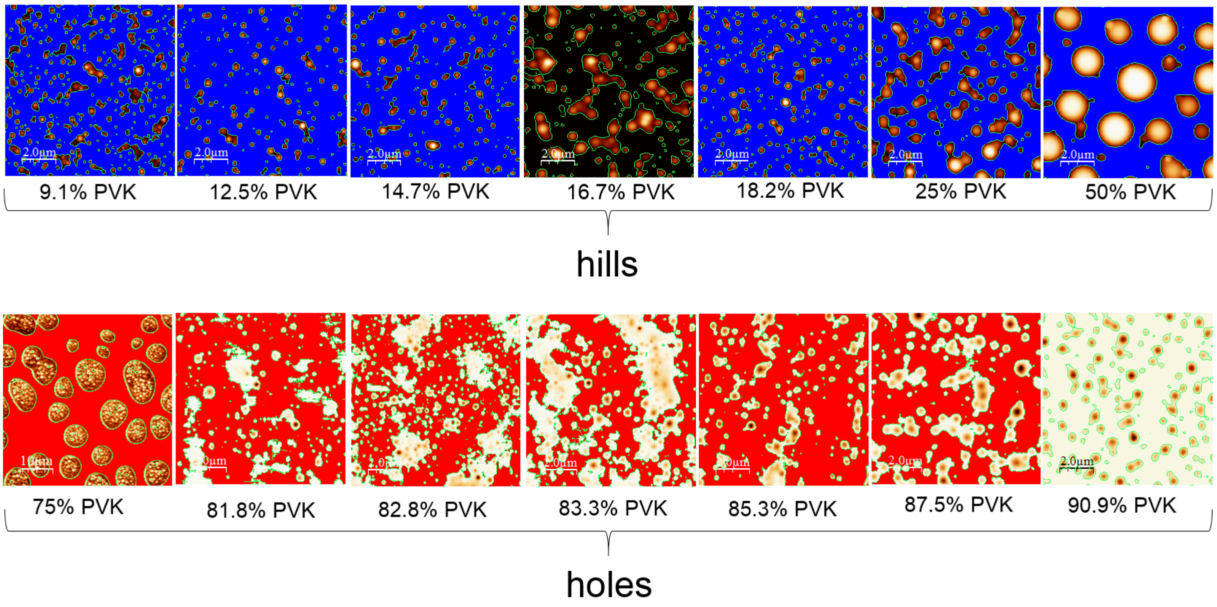

Figure S5. Flooding maps, used for the determination of NND parameters. The first row shows the position of hills, and the second row shows the position of holes.
